# Effect of *Rhodiola rosea* Rhizome and *Punica granatum* Fruit Peel on the Metabolic Processes and Physiological Activity of Rats Fed with Excessively Fat Diet

**DOI:** 10.17113/ftb.61.02.23.7913

**Published:** 2023-06

**Authors:** Maryna Lieshchova, Viktor Brygadyrenko

**Affiliations:** 1 Department of Anatomy, Histology and Pathomorphology of Animals, Dnipro State Agrarian and Economic University, Sergiy Efremov St., 25, 49000 Dnipro, Ukraine; 2 Department of Zoology and Ecology, Oles Honchar Dnipro National University, Gagarin Av. 72, 49010 Dnipro, Ukraine

**Keywords:** high-fat diet, body mass increase, obesity correction, golden root rhizome, pomegranate peel, аtherogenic index of plasma

## Abstract

**Research background:**

*Rhodiola rosea* (golden root) and *Punica granatum* (pomegranate), as well as a number of other species of medicinal plants, exert an array of biological effects: adaptogenic, antioxidant and anti-inflammatory. However, there are not enough contemporary studies of their influence on metabolic processes, especially in cases of imbalanced diet. Lipid dysregulation is the main reason for many diseases, including obesity, cardiovascular disorders, non-alcoholic fatty liver disease, hypertension, atherosclerosis and insulin resistance. Recently, a growing amount of evidence has suggested the positive effects of certain natural nutrients on lipid metabolism. The work aims to define the general effect of golden root rhizome and pomegranate peel on physiological activity and metabolic processes in model animals fed with excessively fat diet. This study is relevant for the development of drugs and food additives for treatment and prophylaxis of metabolism disorders.

**Experimental approach:**

In a 30-day experiment, we determined the effect of golden root (*Rhodiola rosea* L.) rhizome and pomegranate (*Punica granatum* L.) peel on the physiological activity and metabolic processes of 24 laboratory rats consuming a high-fat diet. The physical activity was evaluated according to the mass gain of animals and change in the relative mass of the internal organs, and also the functional conditions of the central nervous system, as demonstrated by the indicators of the locomotor activity and emotional status, determined in the open field test. The influence on the metabolic processes was revealed by biochemical and clinical blood analyses.

**Results and conclusions:**

Body mass of rats fed with golden root (*R. rosea*) reached 125.8 % of the initial body mass; when fed on pomegranate (*P. granatum*), it reached 123.9 %; and the control group reached only 111.5 % of the initial body mass. The rhizomes of *R. rosea* in the diet of male rats during the month of the experiment did not cause significant changes in the relative organ mass, and the pomegranate peel fruits led to a decrease in the thymus relative mass, as well as liver and brain. *R. rosea* rhizomes in the rats' diet led to an increase in the activity of alkaline phosphatase, and also to a decrease in the concentration of urea and urea nitrogen. Diet supplemented with *R. rosea* also contributed to a strong decrease in plasma concentrations of bilirubin and triglycerides (up to 57.0 % compared with the concentration in the control group). The rhizomes of *R. rosea* contributed to an unreliable decrease in the atherogenicity index. The pomegranate peel also greatly increased alkaline phosphatase activity and reduced plasma triglyceride concentrations. In addition, in rats consuming the peel of *P. granatum*, blood glucose concentration decreased. Under the influence of *P. granatum*, a strong increase in the atherogenic index of plasma (up to 518.6 % of the control) was observed because of a decrease in the concentration of high-density lipoproteins (up to 57.1 %) and a simultaneous elevation of the concentration of low-density lipoproteins (up to 158.3 % of the control). Open field test between groups remained without significant changes.

**Novelty and scientific contribution:**

The results indicate that the rhizome of *R. rosea* and peel of *P. granatum* are safe as food additives to high-fat diet and did not cause pathological changes and side effects, and at the same time significantly influence the metabolic processes (lipid and carbohydrate). Our study theoretically substantiates the use of *R. rosea* rhizome and *P. granatum* peel for the production of nutraceutical and pharmacological products for the correction of metabolic disorders of people and animals. Doses and periods of their application require further research.

## INTRODUCTION

Adaptogens are a class of herbal therapeutic and nutritive products that promote strength, adaptability and survivability under stress. They have a pharmacological effect on systems like the immune and neuroendocrine, which ensures their conventional use for the therapy of a broad spectrum of diseases, including those accompanied by metabolic disorders (*1*).

One of the best-known and popular traditional medicinal plants used as an adaptogen to treat stress-related tiredness is *Rhodiola rosea* L. (golden root) (*2*). For medicinal purposes, the rhizome of the plant (underground stem) is mainly used, from which about 140 substances have been isolated, among which the most important are salidroside, rosin, rosavin and *p*-tyrosol. Phytochemical examination showed the presence of flavonoids, phenylpropanoids, phenyl ethanol and benzyl alcohol products, as well as terpenoids and cyanogenic glycosides (*3*). Recent research has pointed out that *Rhodiola* and its compound salidroside may hold pharmacological effects that could be used during the therapy of diabetes, since studies have demonstrated that adenosine monophosphate-activated protein kinase (AMPK) and AMPK-related signalling are associated with its beneficial outcomes (*4*).

The main biologically active substance of *Rhodiola* is salidroside, which has numerous pharmacological effects (antidepressant, anticancer, antioxidant, antihyperlipidemic, antidiabetic, anti-inflammatory, immunomodulatory, *etc.*). The therapeutic effect of salidroside in cardiovascular diseases is based on its antioxidant effect (*5*). This substance has a neuroprotective effect, which has been analysed in experimental models *in vitro* and *in vivo* in patients with ischemic stroke; it has been shown that the main mechanisms include antioxidant, anti-apoptotic and anti-inflammatory effects through the regulation of several signalling pathways and key molecules (*6*). Salidroside has antiproliferative activity against breast, ovarian, cervical, lung, liver, stomach, colorectal, bladder, kidney and skin cancers, as well as glioma and fibrosarcoma. *Rhodiola* extracts and salidroside are used to deliver advantageous effects in treatment of cognitive, behavioural and metabolic conditions. *In vivo* and *in vitro* studies have shown that modulation mechanisms of different synergistic paths that regulate oxidative stress, mitochondrial activity, inflammation, autophagy and cell death, as well as AMPK signalling are involved in the improvement of metabolic disorders (*7*). Despite numerous publications on the benefits of this medicinal plant, a review by Ishaque *et al.* (*8*) indicated that studies of the effectiveness of *R. rosea* in the treatment of physical and mental fatigue are controversial. A review by Hung *et al.* (*9*) also showed that *R. rosea* can have a beneficial effect on the physical and cognitive performance, and on particular mental disorders.

Pomegranate (*Punica granatum* L.) is a long-known and useful plant of the family Lythraceae, which is now widely distributed almost all over the world. The therapeutic prospect of pomegranate is broadly cited in the ancient literature and it was also used in different medical protocols for a combination of conditions. The phytochemical composition of fruits is rich in blends (flavonoids, proanthocyanidins, ellagitannins, vitamins, lipids, organic acids and mineral salts), which are of outstanding biological and nutraceutical value. Due to this reason, attraction to the pomegranate as an object of study by many research groups has increased over the years, especially in the pharmaceutical sector (*10*). Pomegranate (*P. granatum* L.) by-products such as arils, peel, membrane and seeds are a good source of phytochemicals (ascorbic acid, anthocyanins, punicalagin, quercetin, gallic and ellagic acid, *etc.*) with high antioxidant activity, thus providing health benefits (*11*).

Pomegranate peel contains a significant amount of phenolic compounds, such as hydrolysable tannins (gallic and ellagic acids, punicalagin and punicalin) and flavonoids (anthocyanins and catechins), which are important for its biological activity (*12*). Punicalin is the main antioxidant found in pomegranate peel in abundance. Polyphenols from pomegranate not only have a powerful antioxidant ability but also suppress the growth of pathogenic bacteria (such as *Vibrio cholera*, *Pseudomonas aeruginosa*, *Staphylococcus aureus*, *Bacillus cereus* and *Escherichia coli*) and fungi (such as *Aspergillus ochraceus* and *Penicillium citrinum*) (*13*).

Ellagic acid is a well-known antioxidant that can also optimize the lipid profile and improve lipid metabolism, change the concentration of anti-inflammatory mediators and reduce nuclear factor activity, which plays an essential role in the neuroprotective, antiatherogenic and anti-inflammatory impacts of this substance (*14*). The role of pomegranate in relation to endothelial dysfunction and cardiovascular disease is summarized in a review by Delgado *et al*. (*15*): pomegranate components such as flavonoids, tannins, phytoestrogens, anthocyanins, alkaloids and others showed an advantageous effect on the cardiovascular system, improved parameters such as oxidative stress and antioxidant enzymatic system, decreasing the formation of reactive oxygen species and acting as an anti-inflammatory agent. Because of its antioxidant activity, pomegranate is promising in treating chronic diseases. Components that are synthesized from pomegranate decrease oxidative stress and platelet aggregation, reduce uptake of lipids by macrophages, positively influence the function of endothelial cells, and are engaged in the regulation of blood pressure (*16*). Pomegranate fruit biologically active substances have been shown to have strong enzyme inhibitory effects, either directly or indirectly, in the development of diabetes mellitus (*17*). Pomegranate has demonstrated various effects such as antimetastatic, antiproliferative and anti-invasive on different cancer cell lines *in vitro*, *in vivo* and also in clinical trials (*18*). Due to the presence of anthocyanins, ellagitannins, and hydrolysable tannins in pomegranate fruit, which are responsible for antitumour properties by modulating several signalling pathways, its use is promising as a chemopreventive/chemotherapeutic agent (*19*).

Since many publications indicate the ability of pomegranate fruits to influence lipid metabolism, and recommendations are even given for the usage of the plant in the treatment protocol of obesity (*20*), as well as recommendations for its use as an adaptogen (*1*) and immunomodulatory agent, we assumed that this plant would have the same effect on fat accumulation in the body of animals.

Thus, the main action mechanisms of *R. rosea* and *P. granatum* on vertebrates and human body have been studied; however, the details of the effect of these plant preparations on animals fed excessively fat diet have generally remained unexplored so far. The scientific literature describes individual studies of the effects of the components of these plants on various physiological processes in the body, but there is no holistic understanding of the total effect of all plant components on the onset of developing obesity.

The purpose of this research is to define the general effect of golden root (*R. rosea*) rhizome and pomegranate (*P. granatum*) fruit peel on physiological activity and metabolic processes in model animals fed with excessively fat diet.

## MATERIALS AND METHODS

### Ethics

The selection of experimental animals, study protocols and withdrawal of animals from the experiment was authorised by the local ethical committee of Dnipro State Agrarian and Economic University (Decision No. 3 /20-21 of 20.09.2020). The keeping, nourishment, care of the experimental animals were conducted in agreement with the regulations set forth in the European Convention for the Protection of Vertebrate Animals used for Experimental or other Scientific Purposes (*21*) and in Law of Ukraine on protection of animals from cruel treatment (*22*).

### Animals and diets

In the experiment, 24 outbred laboratory male rats were used, divided into three groups: two experimental and one control, 8 animals in each, 1.5 months old with a mean mass of (200±10) g. The animals were kept in polycarbonate cages, 4 individuals per cage, indoors (temperature 20–22 °C, relative humidity 50–65 %, light cycle 12 h light and 12 h darkness). From the beginning of the study, all rats received a diet with excess fat content. For this, 15 % sunflower oil was added to the typical diet that consisted of a 75 % grain mixture which included corn, sunflower grain, wheat and barley, 8 % root crops (potatoes, carrots), 2 % meat bone meal and 2 % mineral-vitamin complex. Once a day (in the morning), each animal was individually given the appropriate amount of root crops, and was observed to eat it. After that, the animal was placed in a cage for 4 animals and they were given the main food in granule form. The animals in the control group received a diet with excess fat content. The main components of this diet were crushed in a mill (such as grain, meat, bone meal and mineral-vitamin complex), mixed, then sunflower oil was added and granules were made from this mixture (feed granulator GKM-100; Tekhnomashstroy, Cherkasy, Ukraine) at the mass of 4200 g for each group during the whole duration of the experiment. Since in our previous studies of other plant species (*23*) the duration of the experiment was 30 days, to ensure the possibility of comparison, the duration of this experiment was the same. Crops like fresh roots, in the appropriate amount, were additionally given every day. All experimental animals had free access to water and food. The amount of food and water during the experiment consumed by each group was noted.

### Medicinal plants

Medicinal plants were added to the diet of the experimental groups at mass fraction of 5 %. Vegetable raw materials (rhizomes and peel) previously dried and crushed were added at mass fraction of 5 % to the mixture of dried and crushed dried diet components, from which the granules were made. The first group received dried and crushed rhizomes of *R. rosea*, and the second the dried and crushed pomegranate peel. For processing the peel, simpler, more environmentally friendly methods that do not endanger the safety of use and quality of the pomegranate peel are preferable. Therefore, in our study, we used dried and crushed (without heat treatment) pomegranate peel, and not its water or alcoholic extract (*24*). Plants were collected from the Oles Honchar Dnipro National University Botanical Garden, Dnipro, Ukraine (48.4355°N, 35.0431°E). The pomegranate fruits were fragmented and deseeded, the peel was crushed to 2–3 mm fragments and hung to dry in the air for five days at 25 °C. The rhizomes of *R. rosea* were cleaned of soil, rinsed, dried and fragmented with a knife into small pieces no larger than 2–3 mm. The obtained plant material was dried in the air for five days in a dark, dry room at 25 °C. After drying, the fragments of the rhizomes and fruit peel were 1.2–2.0 mm and were not fragmented further.

### Morphometric indicators

Each rat was observed and weighed daily, the total mass gain of the animals and the daily mass gain were calculated. Average body mass per group was calculated weekly for individual animals. Live mass and abdominal volume were determined on the first and thirtieth days of the experiment.

### Functional state of the nervous system

The orientation and physical activity as well as the emotional status of the experimental animals were analysed in the open field test (*25*). The used setup consisted of a quadrangle of 1 m^2^ that was divided into 16 squares which were separated by a non-transparent 20 cm high wall. The study was performed in total quiet with extreme brightness of the area itself. An experimental animal in a previously darkened room was taken from a cage, and then put in the middle of the field. Time of the exposure was 2 min. At the beginning of the experiment, each animal was tested for four days in a row (days 1–4) and at the end of the experiment four days as well (days 26–30). The number of crossed squares (peripheral and central) was calculated to assess physical activity; the number of peripheral (with the wall assistance) and central (without wall assistance) racks to assess orientation activity; and the number of acts of grooming, defecation and urination to assess emotional status (*26*).

### Condition of internal organs

All the animals were euthanised on the day 30 of the study using anaesthesia (80 mg/kg of ketamine and 12 mg/kg of xylazine, intraperitoneally) by total bleeding from the heart. Following the autopsy, the condition of the internal organs was visually evaluated for presence of pathological changes. Sampling of organs and tissues (kidneys, liver, lungs, thymus, spleen, stomach, small and large intestines and heart) was carried out using surgical instruments. The internal organ mass was identified with an accuracy of 10 mg.

### Blood study

The blood was withdrawn from the rats in the process of euthanasia. After the narcosis, the blood of the animals was withdrawn directly from the heart. One test tube was used to collect whole blood (1-1.5 mL) to obtain serum for further biochemical assays. Also, a disposable micro test tube with K2 EDTA microcoagulator (Chengdu Rich Science Industry Co., Ltd, Chengdu, PR China) was used for 0.5-1 mL blood for further automatic estimation of formed blood elements and preparation of smears to develop a leukogram.

Blood serum was obtained by leaving the blood undisturbed for some time and then its centrifugation on a СМ-3М.01 MICROmed centrifuge (200×*g*, 5 min; MICROmed, Shenzhen, PR China). All the assays of biochemical blood parameters were carried out using Miura 200 automated analyser (I.S.E. Srl, Rome, Italy). Also, we acted according to the manufacturers’ manuals for the use of commercial reagent toolkits, produced by High Technology Inc. (North Attleborough, MA, USA), PZ Cormay S.A. (Lublin, Poland) and Spinreact S.A. (Girona, Spain).

To evaluate protein and mineral metabolisms in the obtained blood serum, we determined total protein and its individual fraction, albumin/globulin ratio, urea and blood urea nitrogen, creatinine, total bilirubin, total calcium, inorganic phosphorus, ratio of Ca/P, and C-reactive protein. Total protein (g/L) was measured using the Biuret reagent (Cormay Diagnostics, Warsaw, Poland), copper ions of which react with protein in alkaline solution, forming a coloured complex. The colour intensity of the obtained complex was measured spectrophotometrically (sectrophotometer ULAB 102; Ulab, Shanghai, PR China) at *λ*=540 nm and from calibration curve the concentration of protein was determined (*27*). The concentration of albumins (g/L) was measured using a bromocresol green (Cormay Diagnostics), which selectively interacts with albumin in low-acidic media and in the presence of detergent. The developed yellow-green complex was identified spectrophotometrically at *λ*=570–640 nm. Its absorbance is directly proportional to the concentration of albumin in the sample (*27*). Globulins (g/L) were estimated by subtracting the concentration of albumins from total protein concentration. Also, we quantified albumin/globulin ratio. The enzymatic method revealed total bilirubin concentration (µmol/L). We used urease enzyme, which forms a coloured complex by reacting with cholesterol in blood. The colour intensity of the reaction was measured spectrophotometrically at *λ*=490–520 nm, and the absorbance was proportional to the concentration of cholesterol in blood serum (*28*). The urea (mmol/L) was assessed enzymatically. In urease-catalyzed reactions, urea interacts with NADH, the absorbance of which was measured spectrophotometrically at *λ*=340 nm (*29*). At the same time, blood urea nitrogen (mg/100 g) was identified. Creatinine (µmol/L) was determined kinetically, based on the Jaffe reaction with picric acid. Creatinine of blood serum reacts with picric acid in acidic solution, forming a coloured yellow-red complex. The absorption at *λ*=505 nm of the formed complex is proportional to the creatinine concentration in the sample (*30*).

Total calcium (mmol/L) and inorganic phosphorus (mmol/L) were determined using the spectrophotometric method at *λ*=635 nm. Calcium in sample reacted with arsenazo III (Cormay Diagnostics), forming a coloured complex, and phosphorus reacted with ammonium molybdenum (*27*). The indicator of Ca/P ratio is the ratio of total calcium to inorganic phosphorus. The quantification of C-reactive protein (mmol/L) was carried out in relation to turbidity of the solution after latex agglutination with specific antibodies (*31*).

Carbohydrate metabolism was identified according to the concentration of glucose (mmol/L). It was determined by the glucose-oxidase method with consecutive use of glucose oxidase and peroxidase, resulting in the formation of a coloured complex, measured using the spectrophotometric method at *λ*=505 nm (*27*).

The lipid metabolism was evaluated by the following parameters: cholesterol, blood triglycerides, high-density lipoprotein (HDL) cholesterol (mmol/L) and low-density lipoprotein (LDL) cholesterol, and atherogenic index of plasma. Cholesterol (mmol/L) was identified according to cholesterol oxidase, which – in the reaction with cholesterol – develops a coloured complex. Its colour intensity measured spectrophotometrically at *λ*=490-520 nm is proportional to cholesterol concentration in the examined material (*28*, *32*). Blood triglycerides (mmol/L) were quantified enzymatically, using glycerol kinase and glycerol phosphate oxidase, which – as a result of adjacent reaction, in the presence of ATP – forms a coloured complex. The spectrophotometric method was used at *λ*=365-405 nm and the absorption of the complex was proportional to the content of triglycerides in the sample (*33*). HDL cholesterol (mmol/L) and LDL cholesterol (mmol/L) were determined using selective detergents and following identification of the colour intensity of the formed quinine reagent by spectrophotometer at *λ*=600 nm (*34*). We also calculated atherogenic index of plasma.

Changes in the enzymatic activity in blood plasma were monitored in relation to the activities in aspartate aminotransferase (AST, U/L) and alanine aminotransferase (ALT, U/L). For this purpose, we applied the kinetic method based on the Warburg test. The activities of AST and ALT were determined according to the rates of NADH (nicotinamide adenine dinucleotide), and the absorbance was measured spectrophotometrically at *λ*=340 nm (*35*). The indicator of De Ritis ratio was identified by the ratio of the activity of aspartate aminotransferase to the activity of alanine aminotransferase. The activity of alkaline phosphatase (U/L) was determined enzymatically according to the rates of formation of 4-nitrophenol, the absorbance of which was quantified at *λ*=405 nm. The rates of formation of 4-nitrophenol are directly proportional to the activity of alkaline phosphatase (*27*). The activity of γ-glutamyltransferase (U/L) was assessed kinetically according to the breakdown of l-γ-glutamyl-3-carboxy-4-nitroanilide with the formation of 5-amino-2-nitrobenzoate. The rates of its formation were determined spectrophotometrically; absorption at *λ*=365–405 nm is directly proportional to the activity of γ-glutamyltransferase (*27*).

The number of erythrocytes and leukocytes in the stabilized blood of mice was determined using an automatic hematology analyzer MicroCC-20Plus (High Technology Inc). For a leukogram, we prepared blood smears according to Pappenheim and staining was carried out using the Romanovsky–Giemsa method (27).

### Statistical analysis

The tables show results as mean value±standard deviation. Differences between the control and the experimental group values, calculated in the program Statistica v. 7.1 (*36*), were identified using the Tukey’s test (with consideration of Bonferroni correction), where the differences were considered as significant at p<0.05.

## RESULTS AND DISCUSSION

After consuming rhizomes of golden root (*Rhodiola rosea*), the median body mass of the animals reached 125.8 % of the initial mass in the 30 days of the experiment (Table 1). The body mass of animals that had been fed pomegranate (*Punica granatum*) peel increased up to 123.9 % of the initial mass. The control group of animals increased their body only to 111.5 % of the initial mass throughout the experiment. The change in the mass of rats that consumed one of the plants was significant compared with the control group. Thus, both types of plants contributed to the accumulation of animal body mass during one month of the experiment compared to the control group of rats.

**Table 1:** Change in the body mass and fodder consumption of young male rats after *Rhodiola rosea* L. and *Punica granatum* L. supplementation

Parameter	Control^#^	*R. rosea*	*P. granatum*
Change in body mass/(g/day)	0.7±0.3	(1.3±0.3)*	(1.5±0.19)***
Abdominal length/сm	14.0±0.5	13.4±0.7	(15.7±0.5)*
Food consumption/(g/day)	20.09	17.14	19.52
Water consumption/(g/day)	18.42	17.98	19.12

Values are expressed as mean±standard deviation (*N*=8), *t*(experiment)=30 days. ^#^a group of rats fed only a high-fat diet; *p<0.05, **p<0.01 and ***p<0.001 represent significant differences in the same row according to the results of ANOVA with Bonferroni correction

Even more prominent changes in the body mass of animals (Table 1) were noticed *versus* the control group when comparing the average daily mass gain: the addition of *R. rosea* increased body mass gain by 82.3 % and the addition of *P. granatum* by 119.0 %. Interestingly, adding *R. rosea* led to an insignificant decrease in the volume of the abdomen, and *P. granatum* to a significant increase up to 112.2 % compared with the control group.

The taste of animals cannot be reliably judged, but probably the bitter taste of *R. rosea* rhizomes contributed to a decrease in feed intake to 85.3 % of the control, and *P. granatum* peel left the feed intake unchanged (97.2 % of the control group). Water consumption by male rats, when the studied plants were added to their diet, remained at the control group amount (Table 1).

The *R. rosea* rhizomes in the diet of male rats during the month of the experiment did not cause significant changes in the organs’ relative mass (Table 2). The addition of the peel of *P. granatum* fruits to the animals' diet led to a severe reduction of the relative mass of the thymus (up to 48.5 % of the control), liver (up to 79.4 %), and brain (up to 70.2 % of the control) after a month of the experiment.

**Table 2:** Change in the relative organ mass of male rats after *Rhodiola rosea* L. and *Punica granatum* L. supplementation

Organ	*m*(organ)/ %		
Control^#^	*R. rosea*	*P. granatum*
Heart	0.35±0.02	0.37±0.02	0.31±0.02
Liver	4.1±0.2	4.0±0.2	(3.2±0.3)***
Lung	1.0±0.2	1.0±0.2	0.8±0.1
Thymus	0.28±0.05	0.20±0.09	(0.14±0.06)***
Spleen	0.37±0.04	0.36±0.06	0.35±0.07
Stomach	0.67±0.06	0.8±0.2	0.9±0.2
Small intestine	2.6±0.5	2.2±1.0	2.5±0.5
Cecum	0.5±0.2	0.4±0.1	0.5±0.1
Colon	0.37±0.08	0.5±0.1	0.33±0.05
Rectum	0.40±0.07	0.4±0.1	0.31±0.04
Right kidney	0.36±0.03	0.35±0.04	0.31±0.00
Left kidney	0.37±0.04	0.35±0.04	0.31±0.03
Brain	0.87±0.05	0.8±0.1	(0.6±0.2)**

Values are expressed as mean±standard deviation (*N*=8), *t*(experiment)=30 days. ^#^a group of rats fed only a high-fat diet; **p<0.01 and ***p<0.001 represent significant differences in the same row according to the results of ANOVA with Bonferroni correction

The addition of rhizomes of *R. rosea* to the diet of male rats (Table 3) dramatically increased the alkaline phosphatase activity (up to 398.6 % of the control), and also decreased the concentration of urea (up to 71.2 %) and urea nitrogen (up to 71.3 %). Diet supplemented with *R. rosea* also contributed to a strong decrease in plasma concentrations of bilirubin (up to 60.2 %) and triglycerides (up to 57.0 % compared to the control group). The *R. rosea* rhizomes also contributed to an unreliable decrease in the atherogenic index from 1.0±0.4 to 0.8±0.2.

**Table 3:** Change in the blood biochemical parameters of male rats after *Rhodiola rosea* L. and *Punica granatum* L. supplementation

Parameter	Control^#^	*R. rosea*	*P. granatum*
Protein and mineral metabolism			
*γ*(total protein)/(g/L)	77.0±4.9	75.0±5.0	88.3±9.9
*γ*(albumins)/(g/L)	39.6±2.6	40.6±2.0	43.3±3.6
*γ*(globulins)/(g/L)	37.4±3.9	34.4±4.4	45.0±6.8
*N*(albumin/globulin ratio)	1.1±0.2	1.2±0.1	0.97±0.07
*c*(CRP)/(mmol/L)	12.5±5.4	13.6±5.5	25.0±13.5
*с*(urea)/(mmol/L)	6.8±1.0	(4.9±0.5)**	8.6±2.4
*w*(blood urea nitrogen)/(mg/100 g)	13.1±2.0	(9.3±1.0)***	16.4±4.6
*c*(creatinine)/(µmol/L)	63.0±4.4	60.1±6.2	52.6±13.5
*c*(total bilirubin)/(µmol/L)	6.1±1.7	3.7±0.6*	4.9 ± 0.8
*с*(total calcium)/(mmol/L)	2.53±0.09	2.57±0.09	2.3±0.3
*с*(non-organic phosphorus)/(mmol/L)	3.1±0.6	3.7±0.5	3.7±0.9
Ca/P ratio	0.8±0.1	0.8±0.1	0.6±0.1
Carbohydrate and lipid metabolism			
*с*(glucose)/(mmol/L)	7.4±1.0	7.2 ± 0.5	(5.8±0.4)*
*с*(cholesterol)/(mmol/L)	1.3±0.1	1.2±0.2	1.6±0.3
*с*(blood triglycerides)/(mmol/L)	2.1±0.6	(1.2±0.3)**	(1.1±0.4)**
*с*(high-density lipoprotein (HDL) cholesterol)/(mmol/L)	0.6±0.1	0.66 ± 0.09	(0.4±0.3)*
*с*(low-density lipoprotein (LDL) cholesterol)/(mmol/L)	0.5±0.3	0.8±0.1	0.8±0.4
Atherogenic index of plasma	1.0±0.4	0.8±0.2	(5.4±3.4)***
Enzymatic activity in the blood			
*N*(AST)/(U/L)	186±61	178±44	151±101
*N*(ALT)/(U/L)	131±41	153±59	215±84
De Ritis ratio	1.6±0.8	1.4±0.6	0.8±0.6
*N*(alkaline phosphatase)/(U/L)	129±64	(514±134)***	(423±71)***
*N*(γ-glutamyltransferase)/(U/L)	9.3±2.6	11.0±5.6	8.6±4.5

De Ritis ratio=AST/ALT. Values are expressed as mean±standard deviation (*N*=8), *t*(experiment)=30 days. ^#^a group of rats fed only a high-fat diet; *p<0.05, **p<0.01 and ***p<0.001 represent significant differences in the same row according to the results of ANOVA with Bonferroni correction

Pomegranate peel (Table 3) also strongly increased the activity of the alkaline phosphatase enzyme (up to 328.1 % of the control group) and reduced plasma triglycerides (up to 53.7 % of control). In addition, when eating the pomegranate peel, the blood glucose concentration of the plasma of male rats decreased significantly (up to 77.9 % of the control). Under the influence of *P. granatum* (Table 3), there were strong changes in the atherogenic index of plasma (up to 518.6 % of the control) because of the decrease in the concentration of HDL (up to 57.1 % of the control group) and an increase in the LDL concentration (up to 158.3 % of the control group).

Supplementation with golden root rhizomes (Table 4) contributed to a sharp increase in the leukocyte count (up to 189.6 % compared to the control group). The pomegranate peel did not cause significant changes in the cellular composition of the blood (Table 4).

**Table 4:** Change in the complete blood count and leukogram of male rats after *Rhodiola rosea* L. and *Punica granatum* L. supplementation

Parameter	Control^#^	*R. rosea*	*P. granatum*
*γ*(haemoglobin)/(g/L)	126.8±7.0	127.3±12.6	128.0±7.7
*φ*(hematocrit)/ %	40.5±2.7	40.6±3.9	39.6±2.7
*N*(erythrocyte)/(10^12^/L)	6.9±0.3	7.5±0.5	7.2±0.5
*N*(thrombocyte)/(10^9^/L)	339±66	368±78	264±78
*N*(leukocyte)/(10^9^/L)	8.6±1.6	(16.2±6.8)***	9.5±4.8
Leukocyte formula			
*N*(basophils)/ %	0.0±0.0	0.0±0.0	0.0±0.0
*N*(eosinophils)/ %	1.5±0.8	0.3±0.4	0.4±0.5
*N*(eosinophils)/ %	0.0±0.0	0.0±0.0	0.0±0.0
*N*(young neutrophils)/ %	0.0±0.0	0.0±0.0	0.0±0.0
*N*(band neutrophils)/ %	1.2±0.7	0.4±0.7	1.3±1.0
*N*(neutrophils with segmented nuclei)/ %	23.0±8.2	21.3±2.7	20.9±5.7
*N*(lymphocytes)/ %	68.8±8.6	72.6±2.4	73.0±4.4
*N*(monocytes)/ %	5.5±1.3	5.4±2.8	4.3±1.9

Values are expressed as mean±standard deviation (*N*=8), *t*(experiment)=30 days. ^#^a group of rats fed only a high-fat diet; ***p<0.001 represents significant differences in the same row according to the results of ANOVA with Bonferroni correction

Changes in the open field test between groups were not significant (Table 5). An insignificant decrease in the number of urinations was noted in rats that consumed the *P. granatum* fruit peel (to a greater extent) and *R. rosea* rhizomes (to a lesser extent). The peel of *P. granatum* also did not significantly increase the number of faecal boluses in rats. The rhizomes of *R. rosea* and *P. granatum* fruit peel did not lead to changes in the physical and orientation activity of the male rats. *R. rosea* contributed to a non-significant increase in the emotional status, and *P. granatum* to a non-significant reduction in the emotional status of the animals by the end of the experiment.

**Table 5:** Changes in the behavioral characteristics of three rat groups in a 2-minute experiment after *Rhodiola rosea* L. and *Punica granatum* L. supplementation

*N*	Control^#^ 1st–4th day	Control 26th–30th day	*R. rosea* 1st–4th day	*R. rosea* 26th–30th day	*P. granatum* 1st–4th day	*P. granatum* 26th–30th day
Peripheral square visit	28.1±18.0	24.3±14.5	17.9±11.0	16.2±11.5	23.9±14.1	14.4±12.3
Central square visit	1.0±2.3	0.3±1.0	0.4±1.1	0.2±0.8	0.5±2.0	0.1±0.4
Rack in peripheral square	5.5±4.5	3.8±3.1	2.9±2.0	3.3±2.2	4.9±2.5	2.9±2.7
Rack in central square	1.3±1.4	0.7±1.0	0.8±1.4	0.5±1.2	1.0±1.2	0.3±0.7
Grooming act	0.6±0.8	0.6±0.9	0.3±0.6	0.3±0.7	1.5±1.5	0.9±1.4
Faecal boluses	2.2±2.0	2.4±1.6	1.0±1.5	3.0±2.6	3.6±2.2	3.3±2.5
Urination	0.3±0.5	0.4±0.5	0.1±0.3	0.1±0.4	0.2±0.4	0.04±0.2

Values are expressed as mean±standard deviation (*N*=32), *t*(experiment)=30 days. ^#^a group of rats fed only a high-fat diet. There were no significant differences between the groups in all studied parameters (p<0.05 according to the Tukey’s test with Bonferroni correction)

In the present study, the feed intake in the group of rats fed a diet complemented with *R. rosea* was significantly lower and with the addition of *P. granatum* fruit peel slightly lower than of the control. This may be due to decreased palatability of these diets when compared to the diet of the animals from the control group. Taste could be affected by tannins, especially from pomegranate peel (*12*). Cerdá *et al.* (*37*) demonstrated that the extract from pomegranate leaf reduces the feeling of hunger and, accordingly, body mass, and also suppresses the occurrence of obesity and hyperlipidaemia, which is explained by the presence of tannins in the pomegranate, which interact with proteins. At the same time, it is also known that food tannins generally suppress the digestion of endogenous proteins rather than food proteins. Despite the lower average daily feed intake by rats of the experimental groups, the body mass of rats fed *R. rosea* reached 125.8 % of the initial value, of rats fed *P. granatum* it reached 123.9 %, and in the control group it was only 111.5 % of the animal initial body mass.

To assess the toxic effects of various substances (as well as medicinal ones), a sensitive indicator is often used: the mass of organs (*38*). In the present study, the *R. rosea* rhizomes in the diet of male rats during one month of the experiment did not cause notable changes in the organ relative mass or pathological changes, and the peel of *P. granatum* led to a strong reduction in the thymus relative mass (up to 48.5 % of control), liver (up to 79.4 %) and brain (up to 70.2 % of control). Because this indicator made it possible to discover the target organ for the toxicant or to identify signs of endocrine-related influence, it can be assumed that active substances from *P. granatum* peel affect the function of the liver and brain, and activate thymus involution.

Signs of a lipid disorder are atypical cholesterol, triglycerides or free fatty acids concentrations. Hyperlipidaemia and hypercholesterolaemia, and especially high concentrations of plasma low-density lipoprotein cholesterol (LDL-C), are the main inducing factors in the development of atherosclerotic lesions. In this experiment, in animals that consumed a diet with a high-fat content (15 %) for 30 days, both with or without the addition of medicinal herbs, we did not observe changes in the concentration of total cholesterol in blood plasma. At the same time, in the control group of animals that received an excessively fat diet, the concentration of triglycerides sharply increased, and the addition of *R. rosea* and *P. granatum* fruit peel resulted in a considerably sharp reduction of this indicator. The *R. rosea* rhizomes in the rat diet did not lead to such important changes in lipid metabolism. Thus, the concentration of high-density lipoprotein cholesterol (HDL-C) was the same as in the control group animals, and LDL-C concentration did not significantly increase. Against the background of the consumption of pomegranate peel, we observed a sharp decrease in the HDL concentrations with a simultaneous rise in the concentration of LDL-C, which led to a significant increase in the atherogenic index (on average up to 5.41). Brunzell and Ayyobi (*39*) and Rios *et al.* (*14*) indicated that it is the ellagic acid in the pomegranate fruit that reduces the concentrations of total cholesterol and blood plasma triglycerides, but this directly depends on the dose of the substance. Also, a decline in the concentration of total cholesterol and triglycerides in the blood plasma is most strongly induced by pomegranate extract, compared with other fruit extracts. In addition, pomegranate flower extracts reduce concentration of triglycerides, total cholesterol, LDL-C and very-low-density lipoprotein (VLDL-C) cholesterol and increase concentration of HDL-C in rats with streptozocin-induced diabetes, thereby improving metabolic state of the body in diabetes (*40*).

Several pomegranate parts, such as juice, peel, seeds and flowers, have demonstrated blood glucose lowering effects related to the phenolic compounds present in them, putative effects of inhibiting enzymes associated with the metabolism of carbohydrates, stimulating β-cell insulin release, and generally protecting pancreatic tissue (*41*). It is worth mentioning that the hypoglycaemic effect of pomegranate has been determined during *in vivo* studies in rodents with induced hyperglycaemia (*42*). Pomegranate consumption has not been shown to be beneficial in improving glucose and insulin metabolism, and even daily supplementation with pomegranate is not recommended in glycaemic management as a potential therapeutic strategy. In our study, an excessively fat diet and supplementation with *R. rosea* did not cause changes in glucose concentrations, and in rats eating the pomegranate peel, the concentration of glucose in the blood plasma decreased (up to 77.9 % of the control), but at the same time the concentration of glucose in the blood plasma did not go beyond the normal range (*27*).

The excessively fat diet and its supplementation of the test plants also affected the protein metabolism of the rats. The total protein concentration in the blood of the animals consuming a high-fat diet was at the top range of the reference values for this mammalian species (*27*). The addition of pomegranate fruit peel led to an increase in this index both in comparison with the control and in comparison with rats treated with *R. rosea* rhizomes. The increase in the total protein concentration in blood plasma occurred due to the increased content of the globulin fraction, which led to a decrease in the protein coefficient. Addition of *R. rosea* rhizome also lowered the concentration of blood urea (on average to 71.2 % of the control group) and blood urea nitrogen (to 71.3 %). It should be noted that this decrease was also below the reference values for rats (*27*), which may indicate a damaged liver, which is also indicated by a decrease in the total bilirubin level.

The blood C-reactive protein (CRP) level is a marker of an acute inflammatory process in the body. In our study, no significant differences in this blood indicator of rats from the control and experimental groups were found. In a meta-analysis by Sahebkar *et al.* (*43*), five eligible randomized controlled trials did not find conclusive evidence of a significant reduction in CRP when using pomegranate juice, and the effect of pomegranate juice on plasma CRP is autonomous of the duration of its intake.

We found no changes in mineral metabolism in rats. The concentrations of total calcium and inorganic phosphorus in blood plasma in rats of the control and experimental groups were not changed and did not exceed the reference values. Analyzing the enzymatic activity of blood plasma, we discovered that the rhizomes of *R. rosea* in rat diet increased the alkaline phosphatase activity (on average up to 398.6 % of control group values), and the peel of *P. granatum* fruits also significantly increased the alkaline phosphatase activity (up to 328.1 % compared to the control group). At the same time, AST activity indicators did not differ significantly in the control and experimental groups, and there was higher ALT activity than in the control group and this exceeded the reference values during *R. rosea* consumption, as well as during the consumption of *P. granatum* peel.

Despite the fact that many studies indicate that both *R. rosea* and *P. granatum*, and their active substances, in particular, show effects aimed at stimulating the nervous system, reducing anxiety, increasing performance, relieving fatigue, and exhibiting neuroprotective activity (*14*, *44*), in our study, we have not discovered important differences among groups of rats in the influence of these plants on the functional state of the nervous system. At the tendency level, an increase in the animal emotional status was found in the group consuming *R. rosea* and a slight decrease in this indicator under the influence of *P. granatum* in animals receiving the excessively fat diet.

## CONCLUSIONS

The peel of pomegranate (*Punica granatum*) fruits, which had been added to high-fat diet of rats for 30 days of the experiment, caused body mass increment in the animals and changes in their organisms. This is manifested in a sharp decrease in the relative mass of some organs (liver, thymus and brain), significant effect on lipid metabolism (sharp decrease in triglyceride concentration and high-density lipoprotein cholesterol with simultaneous increase in the concentration of low-density lipoprotein cholesterol, leading to fast increase in atherogenic index) and carbohydrate metabolism (decrease in glucose concentration). The rhizome of golden root (*Rhodiola rosea*) in the high-fat diet increased the mass gain as well, but did not have an effect on the relative body mass. This plant led mostly to changes in the functional condition of the liver, manifesting in the decreases in bilirubin concentration, triglyceride concentration, urea and blood urea nitrogen and increase in the alkaline phosphatase activity. The intake of the both plants did not lead to changes in the functional condition of the nervous system (as seen in the results of open field test). In general, the results suggest that the use of these plants in the diet with fat excess does not cause pathological and toxic effects, but at the same time it has an effect on the metabolic processes, making them attractive as components of food additives and pharmaceutical drugs. Further research is needed to evaluate the dosage effect of these plant preparations, as well as the effect of their chemical composition on the health status of model vertebrate species.
